# Theoretical Study on Exciton Dynamics in Dendritic Systems: Exciton Recurrence and Migration

**DOI:** 10.3390/molecules14093700

**Published:** 2009-09-22

**Authors:** Masayoshi Nakano, Ryohei Kishi, Takuya Minami, Kyohei Yoneda

**Affiliations:** Department of Materials Engineering Science, Graduate School of Engineering Science, Osaka University, Toyonaka, Osaka 560-8531, Japan

**Keywords:** exciton, recurrence, migration, master equation, *ab initio* MO, dendrimer

## Abstract

The optical functionalities such as exciton recurrence and migration for dendritic systems, e.g., dendrimers, are investigated using the quantum master equation (QME) approach based on the *ab initio* molecular orbital configuration interaction (MO-CI) method, which can treat both the coherent and incoherent exciton dynamics at the first principle level. Two types of phenylacetylene dendrimers, Cayley-tree dendrimer and nanostar dendrimer with anthracene core, are examined to elucidate the features of excion recurrence and migration motions in relation to their structural dependences. It is found that the nanostar dendrimer exhibits faster exciton migration from the periphery to the core than Cayley-tree dendrimer, which alternatively exhibits exciton recurrence motion among dendron parts in case of small relaxation parameters. Such strong structural dependence of exciton dynamics demonstrates the advantage of dendritic molecular systems for future applications in nano-optical and light-harvesting devices.

## 1. Introduction

Recently, a great deal of attention has focused on the electronic, optical and magnetic functionalities of dendrimers – a new class of supermolecules characterized by branched tree-like architectures – due to their promising applications in future electronics, photonics and spintronics [1,2,3,4,5,6,7,8,9,10,11,12,13,14,15,16,17]. For instance, energy migration in dendrimers is one of hot topics toward a realization of nano-size light harvesting systems, which are candidates for highly efficient optical devices and artificial photosynthetic systems. These attractive functions of dendrimers are predicted to originate in their tree-like structures, together with the high controllability of the structures and sizes. Lots of experimental studies already have been carried out on the excitation energy migration in dendritic molecular systems towards the molecular design of efficient light harvesting systems [14,15,16,17]. Shortreed *et al*. have reported that extended phenylacetylene dendrimers with Cayley-tree structure transport excitation energy absorbing in the periphery region to the central region of the molecule [15]. This energy migration process is found to be highly efficient and directional, in contrast to typical energy transport observed in most of supramolecular antennas in green plants and their artificial polymeric mimics, where the energy transport is partially carried out by random walk and thermal activation due to their disordered structures. They have suggested that such efficient energy migration, described by exciton (electron-hole pair) migration, occurs due to their two structural features: (I) the increase in the lengths of linear legs (π-conjugation) involved in each generation as going from the periphery to the core and (II) the decoupling of π-conjugation at *meta*-branching points (*meta*-substituted benzene rings). These features are predicted to lead to the multistep exciton states with spatially well segmented distribution in each generation: higher exciton states possess dominant exciton distributions in the periphery region, whereas lower exciton states do in the core region [15]. On the basis of such an exciton state structure, several theoretical studies have been performed that have elucidated the mechanism of the exciton migration process in dendritic systems [18,19,20,21,22,23,24,25,26,27,28,29,30,31,32,33,34,35,36]. Using the Frenkel exciton model [18,19,20,21,22,23,24,25,26,27,28,29,30] and molecular orbital (MO) based exciton model [31,32,33,34,35,36], the relationships between the mechanism of energy migration process and tree-like architecture have been clarified: the coupling between exciton and nuclear vibrational states (phonon bath) are essential for the irreversible and directional energy migration in dendritic systems [19,20,21]. Our previous studies have elucidated that the weak exciton-phonon coupling causes the relaxation between exciton states, i.e., energy migration from the periphery to the core, using a dipole-coupled dendritic aggregate model [21,23,24,25] and *ab initio* MO based exciton model [33]. In conclusion, the efficient multistep relaxation (incoherent energy migration) between exciton states turn out to require partial overlaps of spatial exciton distributions between neighboring exciton states, which respectively possess exciton distributions in adjacent generations linked with *meta*-branching points. Namely, the structural features (I) and (II) are found to satisfy these conditions. On the basis of this structure-property relationship, we have investigated the dependence of exciton migration on the variation in excitation energy of core monomer using the aggregate model of “nanostar” composed of phenylacetylenes, and have indicated the possibility of control of energy transfer rate by tuning the energy gap between the core monomer and dendrons (linear legs) in the first generation [25,36].

On the other hand, the coherent dynamics of exciton or electron in super- and supra-molecules have been intensively investigated toward a fundamental understanding of dynamics of excited states and a new development of molecular-based nano-scale devices [37,38,39,40,41,42,43,44,45,46,47,48,49,50]. The coherent excitation by irradiation of laser fields creates superposition states composed of plural excited states, which cause the spatial oscillation of excitation, i.e., exciton recurrence motion. For instance, the exciton recurrence motion between two identical chromophores [2,2’-binaphthyl (BN)] in solution has been experimentally observed by probing the fluorescence anisotropy decay by Hochstrasser and co-workers [40,41]. Yamazaki *et al*. have observed the oscillatory anisotropy decay for anthracene dimer, i.e., dithiaanthracenophane (DTA) in solution, which is longer than that for BN [42,43,44]. Theoretical studies on the mechanism of these phenomena have been performed using the quantum master equation (QME) approach using the dipole coupled aggregate models [45,46] and *ab initio* MO configuration interaction (CI) based QME (MOQME) approach [47,48]. It has been found from these results that the rigid and fixed molecular structure plays an important role in reducing the dephasing rate [30]. There also have been lots of studies on the coherent processes of intramolecular electron transfer. Using a quantum model simulation, Barth and Manz *et al*. have shown the periodic electron circulation in magnesium-porphyrin with cyclic structure, composed of four pyrroline subunits, induced by circular-polarized laser pulse [49]. They have predicted that the chirality of circular-polarized laser pulses can be transferred to unidirectional electron circulation on magnesium-porphyrin. As expected from these results, the dendritic systems composed of rigid building blocks also have a possibility of exhibiting coherent exciton dynamics, e.g., exciton recurrence motion, among their branched structures. 

In the present study, therefore, we clarify the incoherent and coherent dynamics in dendrimers with different architectures using the *ab initio* MO based QME approach with a new exciton picture [47]. We focus on the structural dependence of exciton migration (incoherent process) and exciton recurrence (coherent process) after irradiating a one-mode linear-polarized laser field, and discuss the possibility of controlling these phenomena. The present results will contribute to a novel development of energy transfer/transport and the creation and control schemes of superposition states in dendrimers. 

## 2. Method

In this section, we briefly explain the *ab initio* MO based QME, referred to as MOQME, approach [33,47]. The Hartree-Fock (HF) ground ($|{}^{1}\Psi_{1}\rangle$) and singly excited Slater determinant ($|{}^{1}\Psi_{a}^{r}\rangle$) are employed as the exciton basis {|i〉} (referred to as one-exciton basis) involving the singlet ground (vacuum) $|1\rangle (\equiv |{}^{1}\Psi_{1}\rangle )$ and one-exciton $\left\{|i\rangle \}(\equiv \{|{}^{1}\Psi_{a}^{r}\rangle \}\right)$ (*i* = 2, 3, …, *N*) states. Letters *a*, *b*, ... and *r*, *s*, ... are used to represent the HF occupied and virtual orbitals, respectively. The *α*th electronic state of the molecule, $|\alpha \rangle (\equiv |{}^{1}\Psi_{\alpha }\rangle )$ (which composes the configuration interaction single (CIS) state basis), calculated by the CIS method can be expanded as:

(1) $$|\alpha \rangle =\sum_{i}^{N}|i\rangle \langle i|\alpha \rangle =\sum_{i}^{N}|i\rangle C_{i\alpha }\;(\alpha =1,\;\ldots M)$$

Here, *M* indicates the number of electronic states involving the ground and excited states used in the MOQME approach, and the expansion coefficients $\left\{C_{i\alpha }\right\}$ (referred to as CI coefficients) are obtained by the *ab initio* CIS calculation using the Gaussian 03 program package [51]. The quantum master equation [in the atomic units (h = *e* = *m* = 1)] for the reduced system density matrices $\left\{\rho_{\alpha \beta }\right\}$ in the Born-Markov approximation [52,53]:

(2) $$\frac{d\rho_{\alpha \alpha }}{dt}=-\sum_{\beta }^{M}\Gamma_{\alpha \alpha ;\beta \beta }\rho_{\beta \beta }-F^{l}\sum_{\beta }^{M}\left(\mu_{\alpha \beta }^{l}\rho_{\beta \alpha }-\rho_{\alpha \beta }\mu_{\beta \alpha }^{l}\right),$$

and:

(3) $$\frac{d\rho_{\alpha \beta }}{dt}=-i\left(\omega_{\alpha }-\omega_{\beta }\right)\rho_{\alpha \beta }-\sum_{\gamma ,\delta }^{M}\Gamma_{\alpha \beta ;\gamma \delta }\rho_{\gamma \delta }-F^{l}\sum_{\gamma }^{M}\left(\mu_{\alpha \gamma }^{l}\rho_{\gamma \beta }-\rho_{\alpha \gamma }\mu_{\gamma \beta }^{l}\right),\;(\alpha \neq \beta )$$

where $\mu_{\alpha \beta }^{l}$ indicates the lth component of transition moment between excited states *α* and *β*, and $F^{l}$ indicates the *l*th component of the applied electric field. The first term on the right-hand side of Equation (2) and the second term on the right-hand side of Equation (3) represent relaxation terms involving relaxation factors $\Gamma_{\alpha \beta ;\gamma \delta }$ originating in exciton-phonon coupling. The explicit form of $\Gamma_{\alpha \beta ;\gamma \delta }$ is given by [23,24,33]:

(4) $$\begin{array}{c} \Gamma_{\alpha \beta ;\gamma \delta }=\sum_{k}^{M}\sum_{i}^{N}\left[\delta_{\beta \delta }C_{i\alpha }^{*}{\left|C_{ik}\right|}^{2}C_{i\gamma }^{}g_{\left(i,i\right)}(\omega_{\gamma }-\omega_{k})+\delta_{\alpha \gamma }C_{i\delta }^{*}{\left|C_{ik}\right|}^{2}C_{i\beta }^{}g_{\left(i,i\right)}(\omega_{\delta }-\omega_{k})\right]\\ -\sum_{i}^{N}\left[C_{i\alpha }^{*}C_{i\gamma }C_{i\delta }^{*}C_{i\beta }^{}\left\{g_{\left(i,i\right)}(\omega_{\gamma }-\omega_{\alpha })+g_{\left(i,i\right)}(\omega_{\delta }-\omega_{\beta })\right\}\right]. \end{array}$$

Using the high temperature limit value $g_{\left(i,i\right)}^{0}$, the factor $g_{\left(i,i\right)}(\omega )$ at a temperature T is determined by the following equation:

(5) $$g_{\left(i,i\right)}(\omega )=\frac{2g_{\left(i,i\right)}^{0}}{1+\exp(-\omega /k_{B}T)},$$

which makes the reduced density satisfy the thermal equilibrium condition [28]. From Equation (4), the relaxation pathway and relative relaxation rate of exciton can be evaluated by the relative relaxation factor (RRF), Δg(α→β) [23,24]:

(6) $$\Delta g(\alpha \to \beta )=2\sum_{i}^{N}{\left|C_{i\alpha }^{}\right|}^{2}{\left|C_{i\beta }^{}\right|}^{2}\left\{g_{\left(i,i\right)}(\omega_{\alpha }-\omega_{\beta })-g_{\left(i,i\right)}(\omega_{\beta }-\omega_{\alpha })\right\}$$

where the first and second terms on the right-hand side represent the transition rate from state *α* to *β* and that from state *β* to *α*, respectively. The sign of Δg(α→β) represents the direction of exciton relaxation [positive (negative) value indicates the relaxation from state *α* (*β*) to *β*(*α*)] and the exciton relaxation from state *α* to *β* is fast when the amplitude of Δg(α→β) is large. The terms in wavy-parenthesis in Equation (6) relate to the energy difference between exciton states *α* and *β*, whereas the product of squared CI coefficients, ${\left|C_{i\alpha }^{}\right|}^{2}{\left|C_{i\beta }^{}\right|}^{2}$ represents the weight of having common configurations $\left\{|i\rangle \left(=|\Psi_{a}^{r}\rangle \right)\right\}$ between two exciton states *α* and *β*. These features imply that the relaxation rate and pathway are determined by the state energy difference term weighted by the ${\left|C_{i\alpha }^{}\right|}^{2}{\left|C_{i\beta }^{}\right|}^{2}$ though the driving force of exciton relaxation is the energy difference. 

In the MOQME approach, we first numerically solve the QME, Equations (2) and (3), in the CIS state basis. Second, we convert the system density matrices ($\rho_{\alpha \alpha }$) in the CIS state basis to those ($\rho_{ij}^{\mathrm{ex}}$) in the one-exciton basis {|i〉}. The electron $\rho_{\mathrm{elec}}(r,t)$ and hole $\rho_{\mathrm{hole}}(r,t)$ densities are expressed by [33,47]:

(7) $$\rho_{\mathrm{elec}}(r,t)=\sum_{i(a\to r)=2}^{N}\left[{\left|\psi_{r}(r)\right|}^{2}\rho_{ii}^{\mathrm{ex}}(t)+\sqrt{2}\psi_{a}(r)\psi_{r}(r)\rho_{1i}^{\mathrm{ex}\;\mathrm{real}}(t\right)+2\sum_{j(a\to s)(>i)}^{N}\psi_{r}^{}(r)\psi_{s}^{}(r)\rho_{ij}^{\mathrm{ex}\;\mathrm{real}}(t)],$$

and:

(8) $$\rho_{\mathrm{hole}}(r,t)=\sum_{i(a\to r)=2}^{N}\left[{\left|\psi_{a}(r)\right|}^{2}\rho_{ii}^{\mathrm{ex}}(t)-\sqrt{2}\psi_{a}(r)\psi_{r}(r)\rho_{1i}^{\mathrm{ex}\;\mathrm{real}}(t\right)+2\sum_{j(b\to r)(>i)}^{N}\psi_{a}^{}(r)\psi_{b}^{}(r)\rho_{ij}^{\mathrm{ex}\;\mathrm{real}}(t)].$$

Both the spatial integrations of $\rho_{\mathrm{elec}}(r,t)$ and $\rho_{\mathrm{hole}}(r,t)$ are found to give the excited population due to the orthogonality relation of MOs. The second and third terms of those equations represent the contributions of off-diagonal density matrices, i.e., polarization, between the ground (1) and one-exciton [i(a→r)] configurations, and those between one-exciton configurations, i(a→r) and *j*[a→s for $\rho_{\mathrm{elec}}(r,t)$ and b→r for $\rho_{\mathrm{hole}}(r,t)$], respectively. These second and third terms turn out to be the origin of the polarization of electron and hole densities. The *l*th component of polarization is represented as:

(9) $$P^{l}(t)=\int \rho_{\mathrm{pol}}(r,t)\left(-r^{l}\right)d^{3}r,$$

where $\rho_{\mathrm{pol}}(r,t)$ is a polarization density obtained by [47,54]:

(10) $$\rho_{\mathrm{pol}}(r,t)\equiv \rho_{\mathrm{elec}}(r,t)-\rho_{\mathrm{hole}}(r,t).$$

This equation helps us to clarify the exciton contribution to the (non)linear optical phenomena. For instance, the Fourier transformation of the polarization density provides the dynamic (hyper)polarizability density [54] (by dividing by laser field amplitudes), which can elucidate the spatial contributions of electrons to the dynamic (hyper)polarizability.

## 3. Exciton States of Dendrimers

**Figure 1:**
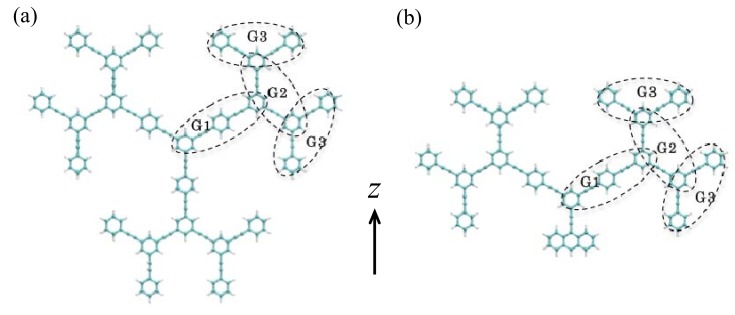
Structures of Cayley-tree dendrimer (a) and nanostar dendrimer with anthracene core (b) as well as coordinate axis.

Figure 1 shows the structures of two types of dendrimers, i.e., Cayley-tree dendrimer (a) and nanostar dendrimer with anthracene core (b), which have three generations (**G1**, **G2** and **G3**). The first generation (**G1**) consists of 1,4-bis-(phenylethynyl)benzene units, while the remaining generations do of diphenylacetylene units. The molecular geometries are optimized under the constraint of *D*_3*h*_ symmetry for (a) and *C*_2*v*_ symmetry for (b) using the B3LYP/3-21G method. 

The low-lying 20 eigenstates (including the ground state) are obtained for these dendrimers using the CIS/3-21G method. In order to reduce the computational effort without loss of qualitative description of exciton dynamics, we restrict the number of MOs included in the CIS active space: (the number of occupied orbitals, that of virtual orbitals) = (23, 23) for (a) and (18, 18) for (b), which consist of π-orbitals. The present one-exciton model is known to qualitatively reproduce the qualitative features of optical absorption spectra for phenylacetylene dendrimers though that tends to provide overshot excitation energies as compared to experimental values [48].

**Figure 2:**
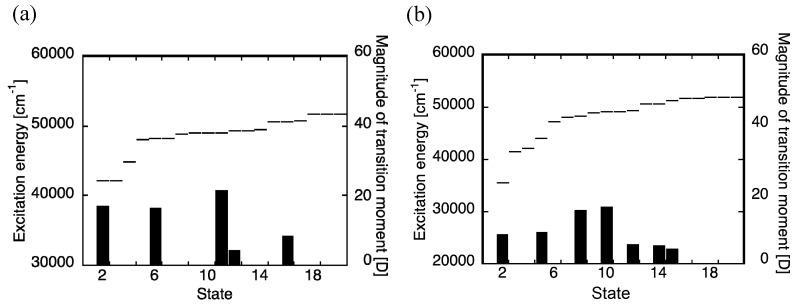
Excitation energies and transition moments (*z*-component) for one-exciton states of Cayley-tree (a) and nanostar (b) dendrimers calculated by the CIS/3-21G method.

**Figure 3:**
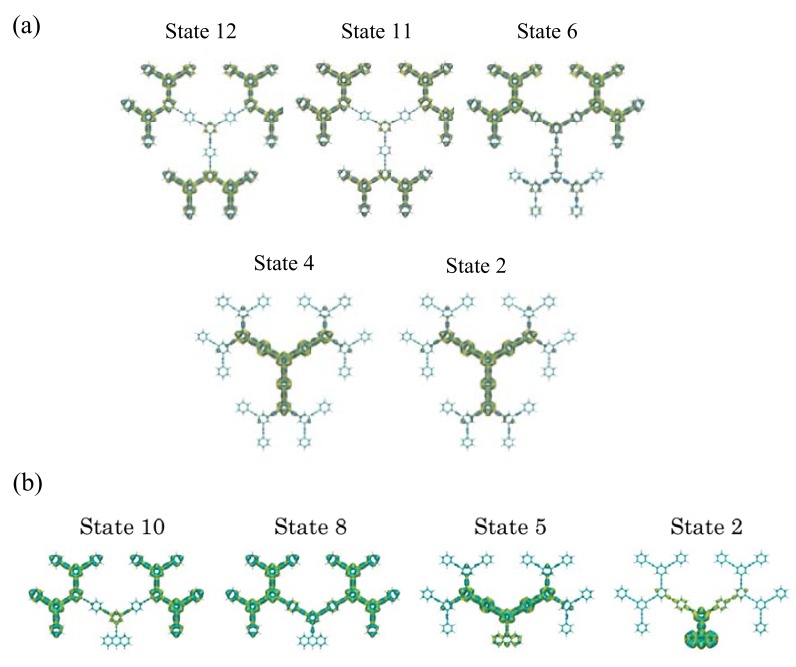
Spatial distributions of exciton [with isosurface 1.0 × 10^-4^ a.u. for electron (yellow) and hole (blue), respectively] in states 12, 11, 6, 4 and 2 for Cayley-tree dendrimer (a) and states 10, 8, 5 and 2 for nanostar dendrimer (b).

Figure 2 shows the one-exciton states of these dendrimers calculated by the CIS/3-21G method. For both systems, we observe multi-step one-exciton states with transition moments from the ground state, in which the excitation energy of the lowest-lying one-exciton state with a transition moment from the ground state (*z*-component) for nanostar (b) is smaller than that for Cayley-tree dendrimer (a). The exciton distributions of several exciton states, which are important for describing the exciton migration processes, for these dendrimers are shown in Figure 3. For Cayley-tree dendrimer (a), states 11 and 12 have dominant exciton distributions in **G3** with smaller distributions in **G2**, state 6 has dominant distributions in **G2** of upper two dendron parts with slight distributions in **G1** and the lower dendron part, and states 4 and 2 have dominant distributions in **G1**. For nanostar dendrimer (b), state 10 has dominant exciton distributions in **G3** with smaller distributions in **G2**, state 8 has dominant distributions in **G2** with smaller distributions in **G1**, state 5 has dominant distributions in **G1** with smaller distributions in **G2** and core, and state 2 has dominant distributions in core with slight distributions in **G1**. It is found from these results that although the features of exciton distributions and the order of excitation energies are similar to each other for both systems, i.e., states 11, 6 and 2 for (a) correspond to states 10, 8 and 5 for (b), respectively, there are some differences: state 2 for (a) is lower than state 5 for (b), and the overlap between states 6 and 2 for (a) is smaller than that between states 8 and 5 for (b) due to the reduction of exciton distributions in **G1** in state 6 and in **G2** in state 2 for (a) than for (b). These differences originate in the replacement of the lower dendron part of (a) by anthracene core for (b). It also turns out that there is a different type of the lowest exciton state for (b) as compared to (a): the exciton distributions of state 2 for (b) are dominantly distributed in core and are slightly penetrated in **G1**. As seen in the next section, these differences in exciton distributions and relative energies cause the significant difference in exciton (energy) migration speed. 

## 4. Exciton Migration Dynamics

**Figure 4:**
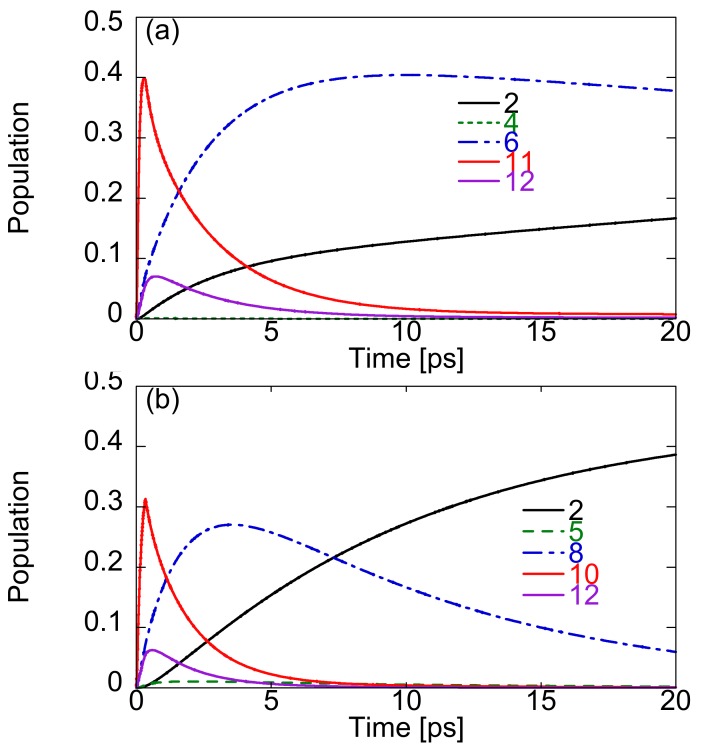
Time evolution of population of one-exciton states primarily contributing to exciton migration process for Cayley-tree (a) and nanostar (b) dendrimers.

**Figure 5:**
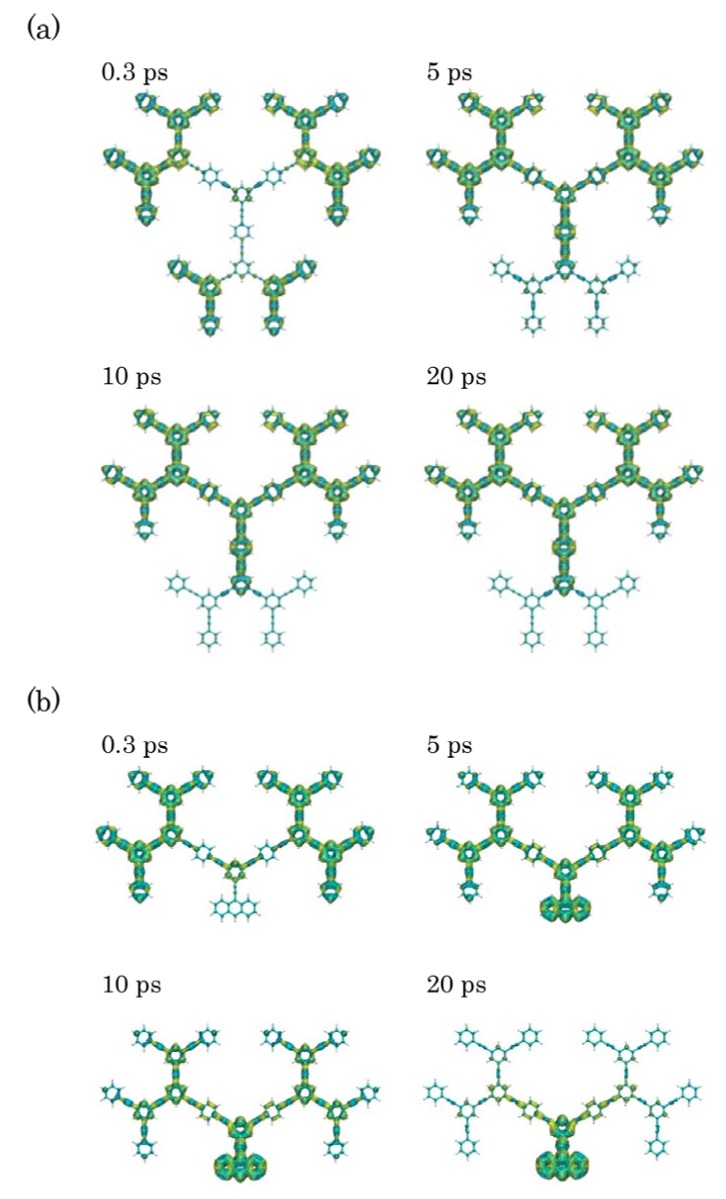
Variation in spatial exciton distributions (with isosurface +5.0 × 10^-5^ a.u. for electron (yellow) and hole (blue), respectively) at times 0.3 ps, 5 ps, 10 ps and 20 ps for Cayley-tree (a) and nanostar (b) dendrimers. The applied external field is cut off at 500 optical cycles (~ 0.34 ps).

Using the one-exciton states obtained by the CIS calculation, the time evolution of exciton population is performed by numerically solving Equations (2) and (3). The temperature *T* is set to 300 K. We choose 200 cm^-1^ for the high temperature limit factor ($g_{\left(i,i\right)}^{0}$), which reproduces the experimental and theoretical migration rates semi-quantitatively previously obtained for similar dendrimers [15,22]. In order to create exciton distribution in the periphery regions, we apply the continuous wave (cw) laser fields, which are in resonance with state 11 (48,990 cm^-1^) for Cayley-tree dendrimer (a), and state 10 (49,064 cm^-1^) for nanostar dendrimer (b). After the irradiation of the laser field during 500 optical cycles (≈ 0.34 ps), we observe exciton migration from the periphery to the core as shown in Figure 4. It is found for Cayley-tree dendrimer (a), the exciton population created dominantly in state 11 rapidly decreases after cutting off the laser field, while the exciton population in state 6 rapidly increases, attains the maximum, and then slowly decreases, while the exciton population in state 2 gradually increases. This leads to the fact that at 20 ps the exciton population in state 2 is still smaller than that in state 6, so that the exciton distribution is not completely concentrated in **G1** but still remains in upper two dendron parts [see Figure 5(a)]. On the other hand, it is found for nanostar dendrimer (b) that the exciton migration to the core anthracene after cutting off the electric field is faster than that for Cayley-tree dendrimer (a): the almost complete exciton population migration from state 10 to 2 through state 8 is observed within 20 ps shown in Figure 5(b). 

**Table 1:** Relative relaxation factors (RRFs) [cm^-1^] [Equation (6)] between exciton states primarily contributing to exciton migration for Cayley-tree (a) and nanostar (b) dendrimers.

(a)	12	11	6	4		(b)	12	10	8	5
11	10.75	–	–	–		10	10.29	–	–	–
6	1.68	1.71	–	–		8	1.76	2.93	–	–
4	0.12	0.14	0.01	–		5	0.69	0.25	0.54	–
2	0.07	0.14	0.02	97.57		2	0.03	0.02	0.01	17.35

In order to further clarify the exciton population dynamics, we investigate the relaxation rate and pathway by the RRF values defined by Equation (6). Table 1 lists the non-zero RRF values between the exciton states primarily contributing to the exciton migration. It is noted that the exciton states contributing to the relaxation pathways in the present symmetric dendrimers are limited to those with the same symmetry as the initial exciton states created by the irradiation of the laser field, i.e., the exciton states with non-zero transition moments from the ground state are allowed to contribute to the relaxation pathways. From the dominant RRF values, the primary exciton migration paths are 11→6→2 for Cayley-tree dendrimer (a) and 10→8→5→2 for nanostar dendrimer (b). The RRF from state 6 to 2 for Cayley-tree dendrimer is 0.02 cm^-1^, which is much smaller than other RRF values and thus is the origin of the slower migration from **G3**/**G2** to **G1** for Cayley-tree dendrimer (a). In contrast, the RRF values form state 8 to 5 for nanostar dendrimer is 0.54 cm^-1^ which is smaller than other RRF values for nanostar dendrimer but is 27 times larger than the RRF value from state 6 to 2 for Cayley-tree dendrimer. This difference in RRF values, which exhibit the exciton migration from **G3**/**G2** to **G1** between Cayley-tree and nanostar dendrimers is the origin of the difference in exciton migration dynamics between these dendrimers. The significant reduction of RRF value in Cayley-tree dendrimer is caused by the small overlap of exciton distribution between the states 6 and 2 due to the slight distribution in **G1** in state 6. On the other hand, for nanostar dendrimer state 8 have relatively large distribution in **G1**, so that the exciton overlap between states 8 and 5 for nanostar dendrimer is larger than that between states 6 and 2 for Cayley-tree dendrimer. As seen from Equation (6), such overlap between exciton states, ${\left|C_{i\alpha }^{}\right|}^{2}{\left|C_{i\beta }^{}\right|}^{2}$, tend to increase the amplitude of RRF value, resulting in the speed up of exciton migration to the core. The difference in the exciton distribution between states 6 (Cayley-tree dendrimer) and 8 (nanostar dendrimer) is predicted to be caused by replacing the lower dendron part of Cayley-tree dendrimer by anthracene core of nanostar dendrimer as mentioned in Section 3. 

## 5. Exciton Recurrence Dynamics

As clarified in the previous section, the exciton migration, which is an incoherent process, strongly depends on the structure of dendrimer through the relative exciton distributions of states involved in the primary migration pathway. Indeed, the Cayley-tree dendrimer is turned out to exhibit slower exciton migration from the periphery to the core than the nanostar dendrimer with anthracene core. On the other hand, there is another important process of exciton dynamics, i.e., exciton recurrence, which is a coherent process and usually can be detected only for a short period due to the phase relaxation caused by the exciton-phonon coupling. From recent experimental and theoretical studies, the rigid structure is found to be necessary for experimental detection of such process. As seen from the results obtained in Section 4, the energy migration process, i.e., population relaxation among exciton states, is slow for the Cayley-tree dendrimer, so that the investigation of the features of exciton recurrence motions as well as their relaxation factor dependences is interesting. In our previous study based on the Cayley-tree dendritic aggregate model including dipole-dipole coupling [45,46], we have observed the exciton recurrence motion among dendron parts even in case of a non-zero relaxation parameter ($g_{\left(i,i\right)}^{0}$ = 10 cm^-1^), and clarified the possibility of controlling recurrence motions by tuning the frequency of the external laser field. The origin of such recurrence motion among dendron parts are clarified to exist in the superposition states composed of near-degenerate exciton states with distributions in the periphery regions, which are created by the initial relevant laser irradiation. In the present Cayley-tree dendrimer, we first consider the non-relaxation case ($g_{\left(i,i\right)}^{0}$ = 0.0 cm^-1^) to elucidate the spatial feature of exciton recurrence motion, and then examine the relaxation effects on the recurrence motion. 

**Figure 6:**
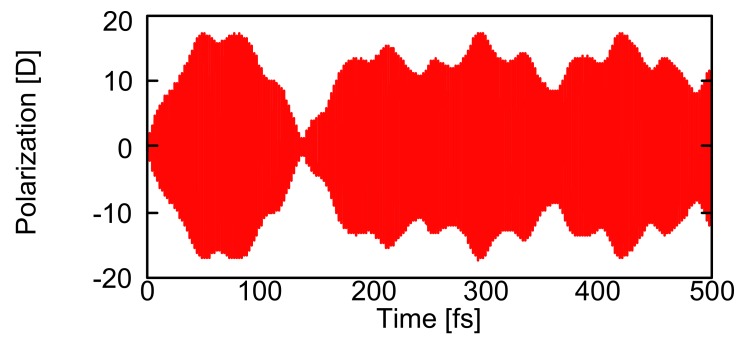
Time variation of polarization (z-component) [D] in Cayley-tree dendrimer in the absence of relaxation parameter ($g_{\left(i,i\right)}^{0}$ = 0.0 cm^-1^).

**Figure 7:**
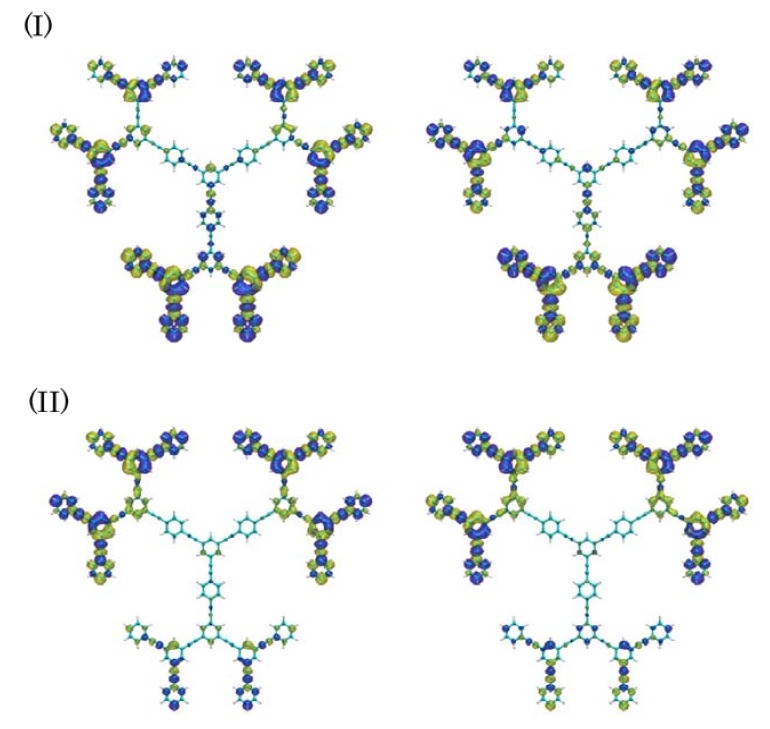
Snapshots of electron and hole densities in Cayley-tree dendrimer in one optical cycle around t = 298.3 fs (I) and 359.3 fs (II) in the absence of relaxation parameter.  The left and right snapshots in one optical cycle around each time show the mutually counter-phase exciton density distributions.  Yellow and blue isosurfaces represent electron and hole density maps with contour values of +5.0 × 10^-5^ a.u., respectively.

As seen from Figure 2(a) and Figure 3(a), near-degenerate states 11 and 12 provide a possibility of creating a superposition state and thus of causing a recurrence motions among periphery regions composed of **G2** and **G3**. The frequency of the external laser field is tuned to 49,200 cm^-1^, which is near-resonant to the near-degenerate states 11 (48,990 cm^-1^) and 12 (49,264 cm^-1^), and irradiation duration is set to 260 optical cycle (~0.25 ps). These conditions are chosen to create a large superposition state between states 11 and 12. It is predicted from the energy difference (~274 cm^-1^) between states 11 and 12 that the recurrence oscillation period is about ~ 122 fs. It is well-known that the exciton recurrence motion is much slower than the polarization oscillation composed of electron and hole distributions, so that we compare the spatial polarization features of exciton distributions at two times having a difference with a half of the recurrence period (~122 fs), i.e., 298.3 fs and 359.3 fs. Figure 6 shows the time evolution of polarization *P* (z-component) and Figure 7 shows the electron and hole distributions giving mutually counter-phase polarizations around 298.3 fs (I) and 359.3 fs (II). Both results are obtained in the absence of relaxation parameter ($g_{\left(i,i\right)}^{0}$ = 0.0 cm^-1^). As seen from Figure 7, the polarization occurs between adjacent bifurcate branches in the outermost periphery regions at both times (I) and (II). On the other hand, dominant spatial exciton distributions are different between (I) and (II): the lower dendron part (**G2** and **G3**) exhibits larger exciton distribution than the upper two parts at (I), while the distributions in the upper two dendron parts are dominant at (II). This indicates the exciton recurrence motion much slower than the polarization period occurs between the upper two dendron parts and the lower dendron part. Such qualitative feature of exciton recurrence motion among the peripheral dendron parts is similar to that observed in the aggregate model [45] though the recurrence motion is more clarified in the aggregate model than in the dendrimer. This difference is predicted to be related to the more delocalized feature of excitons through the *meta*-branching points in dendrimer. 

**Figure 8:**
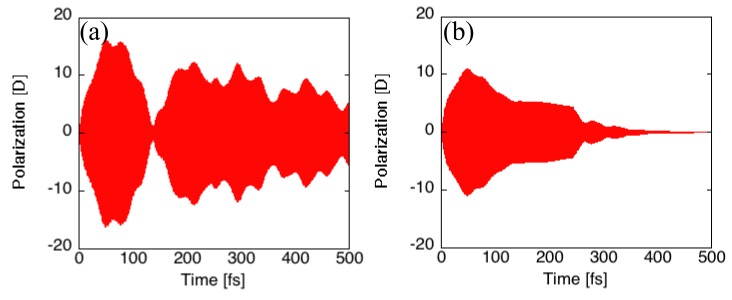
Time variations of polarizations (z-component) [D] in Cayley-tree dendrimer in case of $g_{\left(i,i\right)}^{0}$ = 10.0 cm^-1^ (a) and 100 cm^-1^ (b).

**Figure 9:**
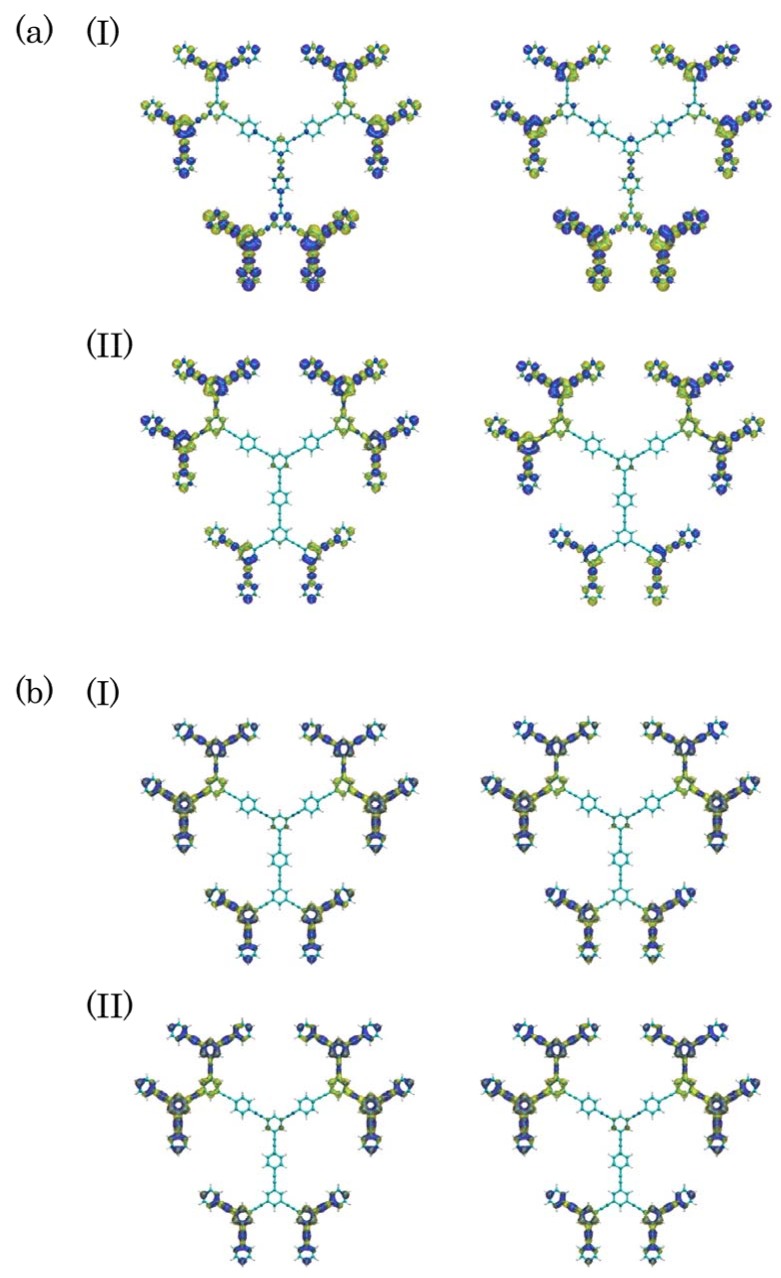
Snapshots of electron and hole densities in Cayley-tree dendrimer in one optical cycle around t = 298.3 fs (I) and 359.3 fs (II) in case of $g_{\left(i,i\right)}^{0}$ = 10.0 cm^-1^ (a) and 100 cm^-1^ (b). See the legend of Figure 7 for further explanation.

Next, we examine the phase relation effects on the exciton recurrence motion using two different relaxation parameters, $g_{\left(i,i\right)}^{0}$ = 10.0 cm^-1^ and 100 cm^-1^. The variations in polarizations and exciton distributions with mutually counter-phase polarizations around two times, 298.3 fs (I) and 359.3 fs (II), are shown in Figure 8 and Figure 9, respectively. Apparently, the decrease in the polarization amplitudes as increasing the time and the tendency is more intensified for larger relaxation parameters. This is found

to be caused by the destruction of the off-diagonal exciton density matrices, which suppresses the polarization, while causes the exciton migration between exciton states. From the comparison between the exciton distributions at the two times for both relaxation parameters, we still detect the exciton recurrence between the two upper and one lower dendron parts in case of $g_{\left(i,i\right)}^{0}$ = 10.0 cm^-1^, though the recurrence amplitude are smaller than the relaxation-free case, whereas hardly detect such behavior in case of $g_{\left(i,i\right)}^{0}$ = 100.0 cm^-1^. This is also understood by the fact that the relaxation time and recurrence period becomes comparable in case of $g_{\left(i,i\right)}^{0}$ = 100 cm^-1^. Of course, the recurrence period depends on the difference between near-degenerate states, but the larger difference between the near-degenerate states cause a difficulty in creating the superposition state by applying the laser field with usual intensity. The experimental realization of exciton recurrence could, however, possible because the sufficient reduction of relaxation factor $g_{\left(i,i\right)}^{0}$ could be achieved by modifying the dendritic structure observed in the present study and by increasing the rigidity of the structure by chemical modifications. 

## 6. Concluding Remarks

In this study, using the *ab initio* MO quantum master equation (MOQME) approach, we have investigated incoherent and coherent exciton dynamics, i.e., exciton (energy) migration and exciton recurrence, in two kinds of phenylacetylene dendrimers, with Cayley-tree and nanostar (with anthracene core) structures, respectively. For exciton migration, nanostar dendrimers exhibit faster exciton migration than Cayley-tree one though both systems give muti-step exciton states with energy gradient from periphery to the core region. This feature is found to be explained by the difference in the energy interval between exciton states and the overlap of exciton distributions between adjacent exciton states, which significantly affect the exciton relaxation factors. Namely, we have found the exciton migration rate has a strong structural dependence on the dendrimers, a features suggesting the possibility of controlling the exciton migration by chemical modifications of dendritic structures. On the other hand, the exciton recurrence motion has been examined in the Cayley-tree dendrimer since this exhibits a relatively slow exciton migration. In the absence of phase relaxation factor, the exciton recurrence motion has been observed between dendron parts, the feature of which originates in the

superposition state composed of near-degenerate exciton states created by irradiating the near-resonant laser field. Because such a recurrence period is around 122 fs in this dendrimer, for small high temperature limit relaxation factor $g_{\left(i,i\right)}^{0}$ ~10 cm^-1^, such exciton recurrence motion appears, while for larger values $g_{\left(i,i\right)}^{0}$ ≥ ~100 cm^-1^, such recurrence disappears and alternatively exciton migration is emerged. From these results, we can predict that the exciton recurrence motion has a possibility of being detected in Cayley-tree dendrimers if the structural fluctuation is sufficiently suppressed, while the efficient and rapid exciton migration is expected in nanostar dendrimer with relevant core molecule [36]. This contribution elucidates that there are strong structural dependences of exciton dynamics, and the dendrimers suited for the coherent and incoherent exciton processes take mutually different structures, e.g., rigid Cayley-tree structure for exciton recurrence, while nanostar (with core molecule) structure for exciton migration. Further investigation of the effects of different variations in dendritic structures and constituent unit structures as well as atom species on exciton dynamics is in progress in our laboratory. 
